# A Comparative Study of Adolescent Social Withdrawal (Hikikomori) in Japan and France

**DOI:** 10.1192/j.eurpsy.2022.603

**Published:** 2022-09-01

**Authors:** Y. Hamasaki, G. Dorard, N. Tajan, T. Hikida, N. Pionnié‑Dax

**Affiliations:** 1 Kyoto Women’s University, Faculty Of Contemporary Society, kyoto, Japan; 2 Universite de Paris, Institut De Psychologie, Paris, France; 3 Kyoto University, Psychopathology And Psychoanalysis Laboratory, Graduate School Of Human And Environmental Studies, kyoto, Japan; 4 Osaka University, Laboratory For Advanced Brain Functions, Institute For Protein Research, Osaka, Japan; 5 EPS ERASME, Child And Adolescent Psychiatry Department, Antony, France

**Keywords:** Hikikomori, mental health, adolescence, Social withdrawal

## Abstract

**Introduction:**

Previously, we conducted a statistical case-control study of adolescent *Hikikomori* patients in Japan using the Parental Assessment of Psychological, Behavioral and Environment Scales. That study did not reveal any pathologies specific to *Hikikomori* patients. On the other hand, environmental factors such as “lack of communication between parents” and “overuse of the Internet” were shown to be significant predictors of *Hikikomori* severity.

**Objectives:**

In this study, using the same methodology as our previous study in Japan, we conducted a case-control study in France. The following questions were examined : (1) whether the pathology of H*ikikomori* patients in Japan and France is the same, and (2) whether the environmental factors associated with the severity of H*ikikomori* are the same in Japan and France.

**Methods:**

Using CBCL and our original scales, we descriptive-statistically compared clinical and subclinical psycho-behavioral characteristics of adolescent *Hikikomori* patients and a control group. In addition, environmental factors that make *Hikikomori* more severe were clarified by multiple regression analysis.

**Results:**

The results showed that there was no difference in the pathology of *Hikikomori* between Japan and France. On the other hand, the statistical predictors of *Hikikomori* severity were “lack of communication between parents and children” and “Lack of communication with the community,” which differed from those in Japan.

**Conclusions:**

Although it is safe to assume that Japanese and French *Hikikomori’*s pathology is generally the same, different strategies may be needed to prevent the onset of *Hikikomori* and to stop it from becoming severe.

**Disclosure:**

No significant relationships.

